# Health of refugees and migrants from former Soviet Union countries in the Russian Federation: a narrative review

**DOI:** 10.1186/s12939-020-01279-0

**Published:** 2020-10-13

**Authors:** Nataliia Bakunina, Artyom Gil, Vitaly Polushkin, Boris Sergeev, Margarita Flores, Igor Toskin, Viktoriya Madyanova, Ruslan Khalfin

**Affiliations:** 1 Institute for Leadership and Health Management, I.M. Sechenov First Moscow State Medical University (Sechenov University), Moscow, Russian Federation; 2 Division of Country Health Programme, WHO Regional Office for Europe, WHO European Office for the Prevention and Control of Noncommunicable Diseases, Moscow, Russia; 3 Moscow Center for Rehabilitation Treatment, Moscow, Russian Federation; 4 Migrant Health Department, International Organization for Migration - Bureau in Moscow, Moscow, Russian Federation

**Keywords:** Migrants’ health, Russian Federation, Non-communicable diseases, Infectious diseases

## Abstract

This narrative review was conducted to synthesize and summarize available up-to-date evidence on current health status, including both non-communicable diseases and infectious diseases, of migrants and refugees from the former Soviet Union countries in the Russian Federation. Epidemiological and sociological studies with one or more determinants of the health, as well as relevant qualitative studies characterizing risk factors, well-being indicators, and lifestyles of migrants and refugees from the former Soviet Union countries in Russia published from 2004 to 2019 in Russian and English languages were included in the review. Despite significant limitations of the available research literature in the field, some patterns in migrants’ health in Russia and issues that need to be addressed were identified. In particular, the syndemic epidemics of communicable and non-communicable diseases, additively increasing negative health consequences, including cardiovascular diseases and chronic digestive system diseases, high rates of sexually transmitted infections and HIV, respiratory diseases and a growing percentage of new tuberculosis cases among migrants from the former Soviet Union countries are all of great concern. Possibly, the burden of these co-occurring morbidities is linked to commonly reported issues among this population group, such as poor nutrition and living conditions, high prevalence of unskilled manual labour, non-compliance with sanitary norms, lack of basic vaccinations, lack of basic knowledge about safe sexual practices and risky sexual behaviour, low healthcare seeking behaviour and limited access to health care. Importantly, these findings may urge the government to increase efforts and promote international collaboration in combating the threat of infectious diseases. Additionally, it was found that migrants had higher levels of anxiety and post-traumatic stress disorder, and those who stayed in the receiving country 5 years or more had a higher level of somatic pathology than those whose stay was less than 5 years. In order to ensure an adequate health system response and fulfil the main Universal Health Coverage principle of “leaving no one behind”, a robust monitoring system of the health status of refugees and migrants and an integrated legal framework for the standardized and more inclusive routine care for this population in Russia is urgently needed.

## Introduction

According to the WHO Global Action Plan, 2019–2023 on promoting the health of refugees and migrants, refugees and migrants are entitled to the same universal human rights and fundamental freedoms, including enjoyment of the highest attainable standard of physical and mental health, equality and non-discrimination [1]. Additionally, WHO Universal Health Coverage (UHC) implicates ensuring all people and communities have access to quality health services where and when they need them, without suffering financial hardship; it is one of the key targets of the 2030 Agenda for Sustainable Development [2]. These strategic priorities are further reinforced in the WHO “triple billion” framework articulated in the 13th General Programme of Work, where the three pillars are (i) advancing UHC, with one billion more people benefitting from UHC, (ii) addressing health emergencies, with one billion more people better protected, and (iii) promoting healthier populations, with one billion more people enjoying improved health and well-being [3]. Meeting these guiding principles has been a huge challenge globally, as well as in the Russian Federation, where, according to different sources, 12 to 22 million foreign citizens reside, with the majority of immigrants being from the former Soviet Union countries [4, 5]. In this narrative review we included sources on communicable and non-communicable diseases (NCDs) published in Russian and English in an attempt to investigate the current health status of refugees and migrants from the former Soviet Union countries in Russia.

Since the dissolution of the Soviet Union in 1991, Russia has become the main center of attraction for hundreds of thousands of migrants and refugees from the former Soviet Union countries due to a number of coinciding political, socio-economic, and cultural factors. First of all, inhabitants of the different former Soviet Union countries often have ties to Russia because of past connections between their states. Large diasporas, use of the Russian language and familiar cultural context across former Soviet republics, geographic location, well-developed transport links in the post-Soviet space, and a visa-free regime for the citizens from the former Soviet Union countries all provided favourable circumstances for migration. Additionally, the mass movement of refugees and internally displaced persons to Russia was caused primarily by a high level of political tension, armed conflicts, and inter-ethnic strife that occurred in former Soviet states throughout the 1990’s and more recently. Finally, the standards of living in the former Soviet Union countries have significantly dropped and poverty has spread rapidly, which forced many people to join the labour market in Russia. By September 2018, the number of labour migrants who indicated the purpose of their entry as “work for hire” reached 3.87 million people and of whom 83% were citizens of the former Soviet Union countries (in August 2017, 4.22 million people indicated their purpose of entry as “work for hire” and 96% of them were citizens of the former Soviet Union countries) [6].

Despite a huge number of migrants and refugees who arrived in Russia in approximately the last 30 years, the Russian governmental system monitors only certain health conditions, mainly infectious diseases, in foreign citizens arriving in the country, in particular, tuberculosis, leprosy, syphilis, and HIV. While some communicable diseases received considerable attention in the research community, a range of issues, such as NCDs or maternal and child health among migrants have not been widely addressed, which is due to lack of both health care access and reporting systems. Although interdependent and interrelated, the legal and regulatory instruments for medical care for migrants and refugees in Russia are rather fragmented, and no integrated legal framework is currently being implemented for the standardized routine care of this population [7]. Furthermore, there have been no regional or national indicators or standards for refugee and migrant health. Notably, many migrants from Central Asia prefer to visit the so-called “Kyrgyz clinics” (private migrant-friendly medical centers founded by migrants from Kyrgyzstan), rather than seek care from municipal health facilities [8]. These clinics, which have been emerging mainly in big cities in Russia since 2010, do not have any system in place for reporting data on their clients’ health to the national governmental surveillance system, and hence a huge proportion of information on the health status of this population is not available [9]. Additionally, there are illegal and unregistered migrants who arrived visa-free as tourists or as businessmen for 90–180 visa-free days but stayed and worked after this period in Russia without any medical check-ups [10]. Therefore, research findings are mostly based on the data of the state monitoring system - Russian Agency for Health and Consumer Rights (Rospotrebnadzor), which accumulates health data of the local population as well as migrants’ health data which is received from the specifically licensed for this purpose clinics (e.g. 24 and 25 state and commercial clinics in Moscow and Saint-Petersburg, respectively). Rospotrebnadzor’s data on migrants’ health is regularly published in a form of non-formalized reports or scientific journal pages, making it the main data source on the topic. However, the reliability of this surveillance data is limited due to several reasons. First of all, this data is collected only for legal migrants showing up for health check-ups in state licensed clinics, leaving behind surveillance of the large proportion of illegal migrants. Both legal and illegal migrants often seek care in other private clinics due to fear of losing their work or residence permit if diagnosed with HIV, tuberculosis, sexually transmitted infections (STIs) or a psychiatric condition. Even if health check-up is performed, the data on the NCD risk factors (alcohol and tobacco use, physical activity, nutrition etc.) is not collected in a structured standardized way and, therefore, is not accumulated centrally in a surveillance system. Moreover, a significant proportion of health check-ups is carried out as a formality without thorough quality assessment of the health status of a migrant. As a result, research data or data for developing evidence-informed policy for promoting health and providing health care to the migrant population in Russia are limited. This narrative review was conducted to synthesize and summarize available evidence reflecting current health status (including both NCDs and infectious diseases) of migrants and refugees from the former Soviet Union countries in the Russian Federation and to identify potential research areas in this field.

## Methods

**Fig. 1:**
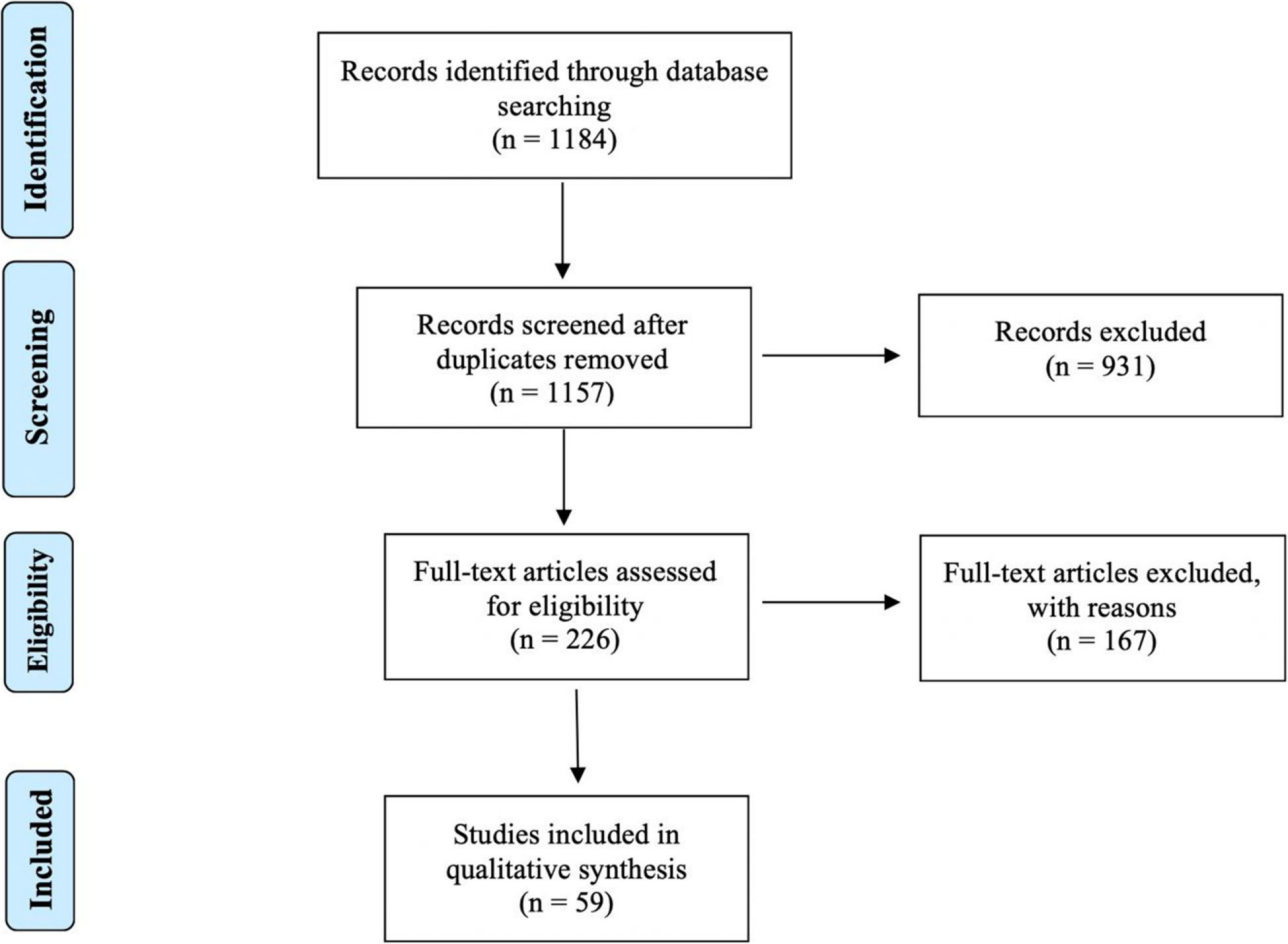
Flowchart of the search results

This narrative review was conducted to address the following research question, “What is the health status of migrants and refugees from the former Soviet Union countries in the Russian Federation?” Since most of the studies on this topic are published in Russian, we performed a comprehensive search in April 2019 that included the Russian databases library.ru, elibrary.ru, CyberLeninka, including the employment of search engines Yandex and Google in Russian language, as well as search of PubMed, Embase, the Cochrane database, the European Health for All Database, OTSeeker, Scopus, Web of Science, the WHO Global Health Observatory, and Peristats. The following keywords and terms were used: migrants* OR refugees* AND Russia* OR Russian Federation* AND non-communicable diseases* OR viral hepatitis* OR mental health* OR tuberculosis* OR infections* OR morbidity* OR detention centers* OR sexual health* OR reproductive health* OR Sexually Transmitted Infections (STIs)* OR syphilis* OR HIV*. To avoid the potential publication bias, comprehensive searching for all eligible studies (regardless of publication status) was performed. The initial literature search yielded a total of 1184 records. After duplicates were removed 1157 records were screened and all articles not containing original research or research on migrants from countries other than former Soviet Union were excluded. 226 full-text articles were assessed for eligibility, of which 59 met the eligibility criteria (see Fig. 1).

We included epidemiological and sociological studies with one or more determinants of health of adult migrants and refugees from the former Soviet Union countries in the Russian Federation from 2004 to 2019. The literature suggests that risk factors, well-being indicators and lifestyles of migrants and refugees may significantly differ from those of local population, so relevant qualitative studies were also included to provide a specific context. Studies on internal migrants and migrants or refugees from countries other than former Soviet Union countries were excluded. However, data on migrants from the former Soviet Union countries were retrieved and included from broad studies that had data on migrants from other countries.

We only included publications in English or Russian. Two authors independently screened the titles and abstracts of the articles and excluded articles not meeting the eligibility criteria. The full text version of an article was obtained if the title and abstract seemed to fulfill the inclusion criteria, or if the eligibility of the study was unclear. The reference lists of the selected articles were also manually searched for any further relevant articles. Due to the nature of the data retrieved for this review, which was any relevant information on the health status of migrants and refugees from the former Soviet Union countries in the Russian Federation and did not include any data on interventions, we expected the publication selection bias to be negligible. With respect to the studies that are heterogeneous in methods and discipline, only narrative synthesis was performed. The data are presented in health topics sections, comparing to indigenous population data when possible.

## Results

### Non-communicable diseases

Multiple factors and complex syndemic interactions during the migration process may lead to a whole spectrum of NCDs. A number of studies investigated social and living conditions of migrants from the former Soviet Union countries in Russia that could potentially exacerbate different pathologies [11–15]. One study conducted in Saint-Petersburg of 150 respondents reported that migrants often live in areas with low-cost housing, which is characterized by overcrowding and poor living conditions, and very few of these migrants possess medical insurance. However, these results were controversial, since no comparison with local population was performed [11]. Another study carried out among 498 labour migrants from Central Asia demonstrated that the main health risk, besides living conditions of the labour migrants, was the nature of their diet, which was characterized by a high intake of low-quality meat products and a low intake of dairy products, fruits, and vegetables [12]. According to a study conducted in 2010 as part of the program “Strategic partnership to promote the rights and empowerment of women migrant workers in Russia”, only one of three migrant women surveyed had adequate housing [13]. Other studies that explored socio-economic conditions of migrant women from the former Soviet Union countries reported a number of challenges often experienced by this population. Almost 100% performed unskilled manual labour and experienced work load pressure or interpersonal conflicts. 35% experienced conflicts with clients, and 3–5% experienced sexual harassment, however the proportion of sexual harassment did not differ from the local population [14, 15]. Although all these components are likely to contribute to development of NCDs, studies suggest that the majority of migrant workers with NCDs manifested their respective symptoms before arriving in the host country, and these pathologies were exacerbated within 6–12 months of their arrival [16].

Neither the epidemiology of NCDs in migrants and refugees from the former Soviet Union countries in the Russian Federation nor a comparison of migrants NCDs morbidity with that of indigenous population received considerable attention in the research literature. However, some data are still available. In order to compare the health status of migrants and indigenous people in Russia in 2009, the Higher School of Economics and “Demoscope” conducted a study based on Russian monitoring of economic situations and health. In this study, information was obtained from 7910 respondents over 18 years old, including migrants from the former Soviet Union and other countries. This group was further disaggregated by the region of origin: those who came from the European former Soviet Union countries - 3.1% (Ukraine, Belarus and Moldova) and those who came from the Asian former Soviet Union countries - 4.3% (Armenia, Azerbaijan, Kazakhstan, Kyrgyzstan, Tajikistan, Turkmenistan, Uzbekistan) as well other countries. Self-assessment of health, the presence of chronic diseases, high blood pressure, excess weight, and smoking were chosen as the main health indicators of migrant and local populations and scores in each indicator were higher in migrants (Table 1) [17]. Compared with local population, migrants from the European former Soviet Union countries were more likely to have high blood pressure (46% versus 32%), have two or more chronic diseases (39% versus 22%), excess weight (66% versus 47%), and were more likely to smoke (39% versus 22%). In all these respects, migrants from the Asian former Soviet Union countries showed similar characteristics as Russian citizens.

**Table 1:** Prevalence of health problems in migrants from the former Soviet Union countries and the native Russian population [17]

	Health problems %				
Indicator	Poor health	High blood pressure	Two and more chronic diseases	Excess weight	Smoking
Never moved	11.9	31.7	21.8	46.6	21.8
Migrants from the European former Soviet Union countries	21.3	46.3	38.9	66.0	38.9
Migrants from the Asian the former Soviet Union countries	11.5	37.5	26.5	49.8	26.5

The study in the Kemerovo region determined the same level of morbidity among migrants (778.31 cases per 1000) and the indigenous population (777.37 cases per 1000 of the corresponding population) based on their treatment demand in governmental clinics from 1999 to 2005. It was established that the intensity of manifestation of circulatory system, digestive system, skin and subcutaneous tissue diseases, as well as diseases of the genitourinary system, was more pronounced in migrant population [18].

Over 3800 diseases per 10,000 migrants were diagnosed in the detention centers of Moscow and the Moscow region in 2016 and 2017, with respiratory diseases being the most prevalent (835.6 cases and 860.2 cases per 10,000 in 2016 and 2017 respectively) and cardiovascular diseases, digestive system diseases, nervous system diseases, muscular and skeletal system and connective tissue diseases present in the top five [16]. Tuberculosis accounted for more than a half of the detected cases of infectious diseases.

### Communicable diseases

#### Tuberculosis

A number of studies reported high prevalence of tuberculosis among migrants [19–23]. At the same time, each year, 26–27% of foreign citizens diagnosed with tuberculosis receive proper treatment in the state clinics of the country [21]. However, a recent study conducted 10 interviews with key informants and revealed that those who tested positive for tuberculosis often disappeared and did not necessarily receive treatment [24]. Migrants are often critically exposed to a large scope of factors associated with tuberculosis: 88% of them are involved in physical labor, 48% have income below a living wage; homelessness and unemployment are widespread [25]. A prospective study conducted in Saint-Petersburg (*n* = 11,484) indicated a significant rise (from 147 hospitalized in 1998 up to 1462 in 2009) in tuberculosis among labour migrants from the former Soviet Union countries over a ten-year period [26]. Another study carried out in Saint-Petersburg reported that tuberculosis prevalence in migrants was 2.6 times higher than that of the ingenious population and presented the segregation of migrants with tuberculosis by the country of origin (Table 2) [23].

**Table 2:** Tuberculosis detection among migrants from the former Soviet Union countries in 2013 in Saint-Petersburg categorized by country-origin [23]

Country	Number surveyed	Detected cases	Per 100,000
Azerbaijan	3000	29	966.7 (648.3-1385.4)
Armenia	3610	10	277.1 (132.9–508.8)
Kirghizia	8964	85	948.1 (758.0–1171.1)
Moldova	8000	53	662.5 (496.6–865.7)
Tadzhikistan	39,949	258	645.8 (570.8–728.0)
Uzbekistan	163,161	752	460.9 (428.9–494.6)
Ukraine	15,783	114	772.3 (596.2–867.1)

Four studies carried out in four regions of Russia (Kaluga (*n* = 39,917), Smolensk (*n* = 500), Khabarovsk (region in the Far East Federal District of the Russian Federation) (*n* = 359) regions and Voronezh (*n* = not indicated)) reported three times higher rates of tuberculosis in migrants in comparison with the local population [19–22]. While data for Voronezh, Far East Federal district of the Russian Federation, and Smolensk were only available for 2014, growing tuberculosis morbidity in Kaluga region from 2011 to 2015 was identified, indicating a considerable increase in the proportion of migrants among detected tuberculosis cases, from 16 to 20.7%. Yet, in 2014 the proportion of migrants in the incidence of tuberculosis was 8.7% in the Far Eastern Federal District, from which 6.5% was in the Khabarovsk region and 8.6% in Voronezh [19, 20]; both were far below the respective figures for Kaluga in 2014. In all regions, infiltrative pulmonary tuberculosis (from 73.3 to 77%) prevailed. In Smolensk 52.2% of migrants with tuberculosis were 25–44-year-old men whereas in the Far East region 86.3% of cases were detected among 20–30-year-old men [20, 22]. Uzbekistan (31.2–36.1% depending on region), Tajikistan (16.7–22.7%) and Ukraine (11.1–19.0%) were identified as the main contributors of tuberculosis-affected migrants in the Russian Federation [19–22]. At the same time, it was documented that in Russia about 3.5–3.7% of tuberculosis cases were reported in foreign citizens [27].

#### Viral hepatitis

At present, screening for viral hepatitis markers is not included in the routine check-up of migrant workers and refugees arriving in Russia [28]. However, the available data on the prevalence of viral hepatitis in the countries of origin (Uzbekistan and Tajikistan) suggest high rates of infection [29]. The data vary, but the frequency of hepatitis C detection in the native population of Russia is between 1 to 2.4% [30]. The data reported in different studies on viral hepatitis prevalence among migrants from the former Soviet Union countries in Russia are summarized in Table 3. These data indicate that hepatitis prevalence diverges across studies: from 4.8 to 37% for hepatitis B, 1.4 to 4.8% for hepatitis C, and from 17 to 24.1% for hepatitis E. However, different statistical methods were applied in data analysis by authors, and two studies did not perform any comparison with local population.

**Table 3:** Rates of viral hepatitis among migrants in Russia by study and year of survey, (%)

Year	Author	Evaluation level	Sample size, migrants/ whole local population	Indicator	Indicator (%), migrants	Indicator (%), local population
**2009**	Mal’ceva et al., 2009 [31]	Khabarovsk city	359 migrants	Viral hepatitis prevalence	HBsAg – 8.6 anti-HBs – 37.7 HBcAg – 1.4 anti-VHA – 78 anti-VHE -IgG – 17	–
**2012**	Kovalevskaya et al., 2012 [32]	Orenburg region	356 migrants, 874 local population	anti-VHE prevalence	24.1	5.37
**2013**	Galperin and Borzunov**,** 2013 [33]	Yekaterinburg	206 female migrants	viral hepatitis B	4.8	–
**2015**	Nechayev et al., 2015 [34]	Saint-Petersburg	4,568,047	delta-coinfection among hepatitis patients	23.0 per 1000	38.3 per 1000
**2017**	Alsalih et al., 2017 [28]	Moscow region	1333 migrants	positive anti-HCV	4.5 (from 3.9 to 4.8 according to the country)	1–2.4%

According to the centre for hygiene and epidemiology of Moscow Region, the proportion of hepatitis C positive migrant workers in Moscow region was 4.5% (3.9% - Ukraine; 4.8% - Moldova and Tajikistan) with 63.6% cases being caused by genotype 1b and 27.3% by genotype 3A [28] in 2017. In 2015, Nechayev reported a rise in the proportion of migrants with Delta hepatitis (hepatitis B + hepatitis D) from 8.8% in 2009 to 37.2% in 2014 in the overall structure of patients with this infectious disease [34].

In Khabarovsk in 2009, hepatitis B was detected in 14.6% of people, hepatitis G - in 5.4% and hepatitis C in 1.4% and hepatitis A in 17% of cases among migrants from the former Soviet Union countries (all of them were citizens of the Central Asian republics) [31]. Overall prevalence of hepatitis E in migrants was reported to be around 17%: 10.87% were female and 89.13% were male; 50% of those infected were 28–38 years old [31]. Hepatitis A was also primarily detected among males (89.13% male, 10.87% female). Half of all positive results were detected in 30–40 years old males, 36.98% among those 18–29 years of age, while 13.04% of hepatitis cases were detected among patients over 40 years [31].

One study that examined awareness of HIV, STIs, hepatitis, and tuberculosis among 150 labour migrants, mostly from the former Soviet Union countries in Saint-Petersburg, found that over a half of the respondents were not aware of transmission routes, symptoms and prevention methods, especially with respect to tuberculosis and hepatitis; women were better informed [35].

#### Other infections

Only one eligible study (*n* = 11,484) on various infectious diseases among migrants and refugees, including tuberculosis and HIV hepatitis, was incorporated in the review [26]. According to the Saint-Petersburg Infectious Diseases Hospital’s data for the period 1990–2009, acute enteritis took a leading position in the structure of the most common infections and accounted for 32.8% of all infections, likely due to consumption of low quality food and non-compliance to sanitary norms [26]. Also, high rates of influenza and other ARVIs (20.3%) as well as the incidence of other viral infections (12.4–31.8%) among migrants has always exceeded those of core population of Saint-Petersburg during 20 years of observation, possibly because of poor living conditions with no heating [26]. On the contrary, in the study conducted in Kemerovo district, a lower level of respiratory diseases among migrants seeking treatment (135,86 cases per 1000) was reported when compared with the indigenous population (185,29 cases per 1000) [18]. In the study conducted in the Saint-Petersburg Infectious Diseases Hospital, 36 people from 11 countries, including Tajikistan and Azerbaijan, had typhoid fever during these 20 years. Malaria was registered in 269 cases over 20 years (from 5 to 15–17 cases per year). Since 1999, Tajikistan (60% of cases) and Azerbaijan (40% of cases) were the main countries of origin for migrants with malaria [26].

The study carried out in Saint-Petersburg found 7 to 80 times higher infection (depending on infection) with brucellosis, *C. burnetiid, S. Typhi, H. pylori,* and *C. diphtheriae* strains in 370 migrants who arrived in St. Petersburg on a work visa than in 320 residents of St. Petersburg with no clear causes for this observation [36].

#### Sexual and reproductive health

All the studies on sexual and reproductive health included in this review were conducted among migrants from Central Asia that arrived in Russia. A recent literature review that aimed to understand what is known about the health of Central Asian female labour migrants in Russia revealed that most of the research in this population has been conducted in the field of reproductive and sexual health [24]. The main findings of this study were that low levels of contraceptive use, increased the risk for STIs and low utilization of sexual and reproductive health services was reported among Central Asian female labour migrants while in Russia. Authors also emphasized that most of the research was performed in males and very little research indicated prevalence rates and epidemiological results for specific health outcomes.

The quantitative sociological surveys on “Tajik migrants, family and reproductive health” and on “Tajik family and labour migration” conducted in Russia and Tajikistan in 2011 that studied the influence of labour migration on family relationships and socio-economic situation in Tajik households provided valuable information on sexual health and behavior of Tajik migrants in Russia. Two hundred fourteen citizens (mainly men) residing in Moscow and Moscow province, whose official or common-law spouse lived in Tajikistan, took part in the survey [37]. Also, 186 Tajik citizens (mostly women), whose spouses worked in Russia during the year preceding the survey or at the time of the survey participated in the study. The Russia-based sample reflected socio-demographic and employment characteristics of Tajik citizens registered by the Federal Migration Service of Russia in 2011. The results of surveys showed that in the 3 years before the interview (2009–2011), 8.9% of Tajik labour migrants in Russia had a history of STIs. At the same time, 11.8% of their family members living in Tajikistan had a history of STIs as well, while only 4.8% of migrant’s partners knew about STIs among their husbands. The results also indicated that during sexual contacts with their spouse, 40.9% of Tajik migrant men used contraception in the majority of cases; 9.6% always used contraception, 17.3% did not use contraception in the majority of cases, and 32.2% never used contraception. 38% of Tajik labor migrants in Russia visited a doctor related to the reproductive health problems at least once. At the same time, only 23% of their spouses in Tajikistan contacted a doctor in regards to their reproductive health [38]. Almost half the surveyed Tajik labour migrants 47.4% had sexual contacts outside their marriage and one-third of them (35%) had contacts with commercial sex workers in Russia [36]. According to data provided by the Tajik research center “SHARK”, 38% of respondents reported that they did not have sexual contacts at all during their stay in Russia, another 22% had sexual contact with casual partners, 11.5% with regular partners (girlfriends), 10% with sex workers, 8% with their wives, 6.5% with a woman who was financially dependent on a man [39]. Notably, 90% of them were married at a time of the survey, but only 5% took their wives with them for the whole period of stay in Russia, another 3% took their wives with them only for a limited period of time [39].

“SHARK” also reported that over the years the number of migrants engaging in casual relationships in Russia almost doubled, whereas the number of those who used commercial sex services did not change, remaining at about 10% [39]. Regarding commercial sex, most often migrants used “girls on call” commercial services, where a woman or a group of women are invited to the place where migrants work or live. In 2010, 52% of migrants who used sex services reported utilizing this type of commercial sex service. Additionally, 16.4% of migrant respondents reported going home to a sex worker, 9% reported visiting brothels, 7% visited massage rooms, 5% paid female employees for sex in the workplace and the rest used saunas, “special apartments,” and cars [39]. The non-commercial sex in which migrants engage was very diverse. This can be sex at the workplace, most often with other female migrants - Moldavians, Ukrainians, Russian, including internal migrants, one-day girlfriends. Moreover, men and women migrants often live all together which facilitated casual sex [40]. This report also indicated that about 70% percent of all migrants used condoms with irregular partners (casual sex, sex workers). However, when a migrant lived with a partner for a fairly long period of time, the partner became “regular” and condoms were not used consistently in these cases [40].

The study conducted by Zabrocki et al. in 2014 examined condom use and intimacy among 400 Tajik male migrants and their regular female partners sampled from bazaars and construction sites in Moscow. Each of the male migrants was married to a woman in Tajikistan and 351 (89%) of them reported also having a regular female partner in Moscow [41]. The study revealed that the length of relationship negatively affected condom use with a regular partner. Another correlate was trust in a partner’s “sexual cleanliness” which was often linked to the partner’s religion and nationality, rather than to awareness and knowledge of HIV risk and protection. In particular, 73% of Tajik male migrants always used condoms with a regular partner who was Christian and only 31% with a regular Muslim partner [41]. An earlier study carried out among 499 male labour migrants from the different former Soviet Union countries in Saint-Petersburg revealed that 30% of them reported multiple female partners in the past 3 months, with condom use ranging from 35% with regular partners to 52% with casual partners [42]. A later study performed among 498 labour migrants from Central Asia in Moscow showed that only half of females who reported multiple sexual partners used condoms regularly [12].

The socioeconomic study on sexual and reproductive health of 1068 migrant females from the former Soviet Union republics carried out by Tyuryukanova et al. in 2011 aimed at investigating sexual and reproductive behavior among women with long-standing partners [13]. The study revealed that 67% of married woman used contraception, however contraception methods were not specified. Among women avoiding contraception, 9% were willing to have a baby, 10% made this decision due to religious or cultural beliefs, 7% followed their partner’s decision, and the remaining 7% refused to answer. Outcomes among women who became pregnant while in Russia (9.1% of questioned migrant women) included 42% having abortions and 32% giving birth in Russia.

The above-mentioned study conducted by Ryazantsev and Akramov in 2011 showed a significant shift towards the reduction of the number of children in the families of Tajik migrants in comparison to the number of children among their parents. For example, only 29.4% of Tajik migrants had three children in the family, while 58.8% of their parents had three children as well. Nearly half of the women with five or more children stated that they would have liked to have fewer children than they actually had, which suggests that some of their births were unwanted [37]. Another study conducted in Kemerovo region found that the birth rate among migrants from the former Soviet Union countries was on average 2.5 times higher than among the indigenous inhabitants [18].

#### HIV/AIDS and syphilis

Migrants can be vulnerable to HIV when practicing risky behaviour under the pressure of financial needs or stress. Furthermore, limited access to health care may prevent them from getting timely advice and help. Despite these risks, official statistics reveals that foreign citizens do not significantly contribute to the HIV epidemiological situation in Russia. Among newly detected HIV infections, migrants constituted between 5.3 to 7.8% [43]. A recent rise in detection of HIV cases in migrants was prompted by the introduction of obligatory HIV testing in migration centers for those applying for work permits. In recent years, approximately 3500 new HIV infections were diagnosed among registered migrant workers in Russia annually, that is, among those who officially applied for a work permit. Notably, in some countries, such as Ukraine, the incidence of HIV is similar to that of Russia and as result, the countries of migrants’ origins play a significant role in the overall structure of HIV incidence in the Russian Federation. Thus, migrants with HIV primarily arrive from Ukraine, Uzbekistan, and Tajikistan. Notably, citizens coming from the Eurasian Economic Union are not required to undergo compulsory HIV testing, and, therefore, no reliable data on these countries are available.

The majority of the studies conducted in Russia used federal reporting forms as a primary source of information, yet the difference in numbers is attributable to different analysis modalities (Table 4).

**Table 4:** HIV detection rate in migrants and the local population per 100,000 in 2012–2016

Year	Author	Evaluation level	Sample size, migrants/ ocal population	Detection rate in migrants	Detection rate in local population
**2012**	Onishchenko, 2012 [44]	Russian Federation	1070 887 / 24,700,000	113.45	251.012
**2012**	Korita et al., 2012 [45]	Far Eastern Federal District	927,839	149.27	165.2
**2014**	Popova, 2016 [46]	Russian Federation	7,400,000	153.5	–
**2014**	Belyakov et al., 2014 [47]	Saint-Petersburg	744,396/ no data	170.0	400.0
**2014**	Sofronov et al., 2014 [23]^a^	Saint-Petersburg	242,611	171.1	-^a^
**2015**	Korita et al., 2015 [48]	Far Eastern Federal District	101,696	99.3	–
**2015**	Lioznov et al., 2015 [49]	Northwestern Federal District	373,382	169.26	–
**2015**	Nechayeva et al., 2015 [27]	Russian Federation	7.4 mln/no data	107.5	315.1
**2015**	Struin, 2015 [50]	Sverdlovsk region	4,395,600	87.1	79.9
**2016**	Belyakov et al., 2016 [51]	Northwestern Federal District	447,034 / 2,285,150	152.5	303.7

^a^ high probability of bias (the data on local population is calculated as a number of HIV-positive per 100,000 members of the population, while data on migrants is calculated for the number of those screened)

Most studies provided consistent data on HIV detection in migrants. Lower detection rates in the Far Eastern Region can be explained by a low proportion of migrants from HIV-epidemic countries, like Moldova and Ukraine [45]. While substantial variability is observed across studies, analysis of the Federal Districts revealed even more incoherence. For example, the low and high estimates for the Far Eastern Federal District were 39.1 and 333.1, respectively [48]. Long-term evaluation of the same region also showed substantial fluctuation in the reported prevalence of HIV among migrants over time: in 2005 there were 116.6 migrants per 100,000 screened in the North-Western Federal District, 273.2 per 100,000 in 2010 and 169.3 per 100,000 in 2013 [49]. The study commissioned in Saint-Petersburg with a sample size of 11,498 people reported 146 HIV-positive migrants with the majority of them being from the former Soviet Union countries during 1980–2009 [26]. Transmission routes were undetermined in 87% of HIV-positive migrants. Some migrants reported heterosexual contacts or drug abuse, but denied homosexual intercourse [47]. In the meantime, some studies reported homosexual contact and commercial sex among the migrant population in the Russian Federation [52–54].

A number of studies conducted formative research among migrants from the former Soviet Union countries in Russia to understand risk factors, risky sexual behavior, and socio-structural barriers to HIV care among subgroups of the migrant population. In particular, male Tajik injecting drug users did not have adequate knowledge about HIV risk through needle-borne infection, had little or no access to formal health service, and were at risk for social marginalization from both Russian society and their own Tajik migrant community [55]. Female migrants from Kyrgyzstan and Tajikistan reported difficulties with acquiring documents for legal status, financial insecurity, discrimination, and sexual harassment, all of which are barriers to HIV prevention [56]. For men who have sex with men from different countries, including the former Soviet Union countries, key risk factors included activity in Moscow sex work, high numbers of partners, inconsistent condom use, avoidance of HIV testing or purchasing false results, as well as the stigma and violence related to homophobia [54]. Results from the study carried out in Moscow, Novosibirsk, and Yekaterinburg that included female migrants from Kyrgyzstan, Tajikistan and Uzbekistan showed that the prevalence of risky sexual behaviour is lower among migrants in comparison with local females. At the same time, migrants were less capable of discussing safe sexual practices with their regular partners, less likely to perform HIV testing, and their access to health care was limited [57]. Thanks to obligatory testing, some data are available on syphilis for migrants entering the Russian Federation.

Although easily cured at early stages, syphilis is one of the most prevalent infections in migrants (Table 5). In the study conducted by Kungurov et al. 16 STIs clinics in Ural, Siberia, and Far East provided official data on syphilis incidence from 2003 to 2009 [58]. The analysis of the data showed that percentage of migrants in the overall syphilis incidence was between 0.2% (in the Tuva Republic) to 36.9% (in Tumen’ region), while syphilis incidence among legal migrants only was 5–30 times higher than the incidence of syphilis in the indigenous population. The same study performed interviews with 34 migrants infected with syphilis and two control groups, one of locals with syphilis, the other of healthy locals. The results indicated that 19% of respondents who were migrants used commercial sex services in comparison with 3 and 0% in control groups. Notably, 37.5% of migrants disappeared after they were diagnosed with syphilis for unknown reasons [58]. The more recent study by Ulitina et al. that aimed to evaluate the epidemiological role in the structure of syphilis incidence in Surgut between 2012 and 2014, indicated 413.3 cases of diagnosed syphilis per 100,000 examined migrants, which was 3.7 times higher than the average in the Russian Federation in the same year [59]. It was also reported that the proportion of migrants in the overall structure of syphilis incidence in Surgut had increased from 4 to 46.4% over 3 years, suggesting half of the people with syphilis had been a migrant in the respective period [59]. This increase could be explained by the introduction of a more specific and sensitive treponemal test for syphilis (enzyme-linked immunosorbent assay) in 2014 instead of non-treponemal test that was used prior.

**Table 5:** Studies reporting syphilis prevalence in migrant and local populations in regions of Russia, (per 100,000, %)

Author	Screening region	Sample size, migrants/whole local population	Indicator (per 100,000) migrants	Indicator (%) local population
Kungurov et al., 2010 [58]	Ural, Siberia, Far East	126,279	142–1877	28.4–62.9
Struin, 2015 [50]	Sverdlovsk region	–	429.2	65.6
Ulitina et al., 2015 [59]	Surgut city	17,151	413.4	45.5

#### Mental health

Migration, either voluntary or forced, is a major stressful event for individuals and families and, therefore, is a prominent risk factor for exacerbation of mental disorders [60]. Additionally, migrants are constantly exposed to major stressful factors at their workplaces, such as higher classes of hazards, low levels of professionalism, violations of safety rules, and high labour intensity [14].

The socio-psychological aspects of migrant life were assessed by Ovchinnikov in 2014 and Khodzhiev in 2017. Ovchinnikov investigated socio-psychological adaptation style, personal and situational anxiety levels of 50 migrant and 50 local medical students in Novosibirsk using relevant scales and surveys. The study showed that migrant students scored significantly higher on such scales as “Interactivity,” “Alienation,” “Depression,” and “Nostalgia,” whereas they scored significantly lower on the “Adaptability” scale when compared to local population [60]. Migrant students found themselves in a quite different social situation where they had to adapt to new social environments. Being aliens, they had to cooperate with their compatriots (showing high level of interactivity), however when no compatriots were around they were more likely to be depressed and nostalgic. Another study that investigated psychological adaptation of migrants from Uzbekistan in Russia using a national validated psychological test reported signs of better adaptation among migrants who wanted to obtain permanent residence (*n* = 148) when compared to migrants willing to return to their home country (*n* = 62) [61]. One study conducted in Moscow and Moscow region revealed increased levels of personal anxiety in 50–75% of labour migrants in comparison with local workers (*n* = 219, divided into 5 groups, 2 of which consisted of labor migrants) [14]. This study also investigated the distress caused by sexual harassment among migrants: almost 61% of men and 49% of women had some signs of post-traumatic stress disorder or related psychological trauma [14].

The study that analysed self-reported psychological well-being in 115 Russian citizens and 90 migrants in Russia showed that migrants demonstrated a lower level of satisfaction in theirs relationship with their spouse(s), children, and friends. However, no statistically significant differences in the indicator of subjective well-being were found between Russians and migrants [62]. The study carried out in Valdivostok explored adjustment disorders in population groups at a high risk for developing a mental disorder (400 police officers that had returned from hot spots, 300 jobless miners, and 100 migrants from Central Asia). Individual and social disadaptation was the highest in migrants, followed by jobless miners, and then police officers [63].

No studies assessing depression, anxiety, or psychotic disorders were found while conducting this review, however many different studies from other countries suggest that in refugee and migrant groups, the prevalence of depression ranges from 5 to 44% and anxiety ranges from 4 to 40%, compared to a prevalence of 8–12 and 5% respectively in the general population [64].

## Discussion

This narrative review provides an overview of the current knowledge on health status and health risks faced by migrants and refugees from the former Soviet Union countries in the Russian Federation. Importantly, this study revealed significant limitations of the available research literature with respect to the health of migrants in Russia, which did not allow us to draw generalizable conclusions. However we believe that highlighting these limitations may shed light on what data are available and where significant research gaps are present in this field. First of all, the review revealed that over half of the publications covered three main topics: HIV, tuberculosis and other infectious diseases. While the exact reasons for the predominance of the publications focusing on these health issues are not clear, this could be due to a range of factors, such as national research agenda and funding opportunities, health emergencies and disease outbreaks, mandatory screening for particular infectious diseases in the country and hence available data on these conditions. Thus, the source of data in the reported studies was mainly governmental statistics accounting for migrants who are legally residing in the country and therefore, excluded all illegal migrants or those who could come to Russia visa-free. Therefore, more reliable sources of data on health conditions in migrant population in Russia are warranted and for example population based surveys could be a valuable source of information.

Notably, the proportion of migrants who were actually screened ranges from 28 to 75% depending on the region and year which is still higher than in the local population [48]. The remaining studies provided primarily self-reporting data, which apart from having general bias issues, tended to include particular population subgroups: those who lived in big cities and were fluent in the Russian language, and thus were able to take part in the survey. Second, the majority of the studies did not compare health indicators of migrant and local population using similar methodology. Therefore, it is not clear whether obtained results reflect the situation in the country in general or indicate a particular health profile of migrants from the former Soviet Union countries living in Russia. Third, most of the studies did not provide information on the legal status of migrants, which dramatically influences access to medical care and living conditions. Overall, the potential contribution of migration to the spread of communicable diseases that influence the sanitary and epidemiological situation in Russia remains uncertain. Clearly, in order to assure adequate health system response, the robust monitoring system of the health status of refugees and migrants in Russia is required.

Nevertheless, some patterns in migrants’ health in Russia and potential problems that need to be addressed were identified in the review. In particular, poor sexual and reproductive health outcomes, including high rates of STIs, including syphilis and HIV, high prevalence of sexual contacts outside marriage, utilization of commercial sex services, inconsistent use of condoms, and lack of basic knowledge on safe sexual practices among migrants from the former Soviet Union countries, are of great concern. Additionally, while overall tuberculosis morbidity in Russia decreases, the percentage of migrants among the new cases grows, which should urge the government to increase its international efforts in combating the tuberculosis threat. On the other hand, such results may be linked to specific detection patterns: tuberculosis in migrants is usually found following a mandatory X-ray during routine check-ups, whereas the local population is normally diagnosed with symptomatic tuberculosis. In particular, foreign citizens and those who do not have citizenship are considered to be at high risk for tuberculosis. Thus, when they apply for a temporary or permanent residence permit, citizenship or work permit in the Russian Federation migrants are required to provide confirmation of their tuberculosis status as well as to be routinely screened for tuberculosis once a year. Following the examination only migrants who come to the specialized medical institution with tuberculosis are registered and others either leave the country or reside illegally. On the other hand, early diagnosis of tuberculosis in migrant population may lead to timely and more effective treatment reducing mortality due to this condition, thus causing a health-selection effect. However, lack of access to quality medical care among migrants, including tuberculosis care in Russia, and a policy of deporting such migrants back to their home countries for further treatment, diminishes the benefits of the health-selection effect. Furthermore, migrants often lack basic vaccinations and have a higher rate of infectious diseases. Although migrants’ mental health studies in the Russian Federation are very scarce, in our review we found risk factors such as stress, economic hardship, poor living conditions, and social isolation experienced by migrants from the former Soviet Union countries, similar to those identified in European countries, where it is suggested that migrants are more prone to certain mental disorders [65]. These findings emphasize the need for structured integration and assimilation programmes for foreign citizens (compatriot communities support can be very helpful, especially in the beginning) as well as increased exchange and collaboration between mental health specialists and social workers across the former Soviet Union countries. Still, the health of migrants is not always worse in comparison to local population, possibly because those individuals who decide to undertake an endeavour such as migration to a different country are initially healthier. This could be also explained by a well-documented in various research phenomenon known as “healthy migrant effect”, when recent immigrants are on average in better health than the native born, although mortality and morbidly indicators are higher in the immigrants’ home countries. While there is no single cause for this effect, the most studied interpretations which are interrelated include positive self-selection in terms of the socioeconomic and health characteristics of the migrants, health screening system in the host country, premigration healthy behaviour, “cultural buffering” (delay in the adoption of unhealthy behaviours of the local population), return of the less healthy migrants’ home and other theories [66–70]. However, another explanation of this effect, which is particularly relevant for Russia, is that immigrants having health problems, especially infectious diseases like tuberculosis, HIV or STIs, are deliberately avoiding contacting official health system because of the fear of losing job in Russia and being deported to their home countries, if these diseases are diagnosed during compulsory health check-up. At the same time, migrants who stay in the receiving country 5 years or more have a higher level of somatic pathology than those whose stay fewer than 5 years [16].

## Conclusion

In this narrative review we aimed to map out available literature on the health status of migrants and refugees from the former Soviet Union countries in the Russian Federation. The review highlights major knowledge gaps in the field as well as provides a snapshot of coverage by and access to health care across migrant population from the former Soviet Union countries in Russia. Development of a reliable monitoring and evaluation system and integrated legal framework for equitable prevention, treatment and care services for migrants in the Russian Federation is urgently needed for ensuring the main UHC principle of “leaving no one behind”.

## Data Availability

Data sharing is not applicable to this article as no datasets were generated or analysed during the current study.

## Abbreviations

NCDs: Non-communicable diseases
STIs: Sexually transmitted infections
UHC: Universal Health Coverage
